# Patterns of environmental variance across environments and traits in domestic cattle

**DOI:** 10.1111/eva.12924

**Published:** 2020-03-06

**Authors:** Mads F. Schou, Torsten N. Kristensen, Ary A. Hoffmann

**Affiliations:** ^1^ Department of Chemistry and Bioscience Aalborg University Aalborg East Denmark; ^2^ Department of Biology Lund University Lund Sweden; ^3^ School of BioSciences Bio21 Institute The University of Melbourne Melbourne VIC Australia

**Keywords:** cattle, directional selection, environmental variance, evolution, selection intensity, stabilizing selection

## Abstract

The variance in phenotypic trait values is a product of environmental and genetic variation. The sensitivity of traits to environmental variation has a genetic component and is likely to be under selection. However, there are few studies investigating the evolution of this sensitivity, in part due to the challenges of estimating the environmental variance. The livestock literature provides a wealth of studies that accurately partition components of phenotypic variance, including the environmental variance, in well‐defined environments. These studies involve breeds that have been under strong selection on mean phenotype in optimal environments for many generations, and therefore represent an opportunity to study the potential evolution of trait sensitivity to environmental conditions. Here, we use literature on domestic cattle to examine the evolution of micro‐environmental variance (CV_R_—the coefficient of residual variance) by testing for differences in expression of CV_R_ in animals from the same breed reared in different environments. Traits that have been under strong selection did not follow a null expectation of an increase in CV_R_ in heterogenous environments (e.g., grazing), a pattern that may reflect evolution of increased uniformity in heterogeneous environments. When comparing CV_R_ across environments of different levels of optimality, here measured by trait mean, we found a reduction in CV_R_ in the more optimal environments for both life history and growth traits. Selection aimed at increasing trait means in livestock breeds typically occurs in the more optimal environments, and we therefore suspect that the decreased CV_R_ is a consequence of evolution of the expression of micro‐environmental variance in this environment. Our results highlight the heterogeneity in micro‐environmental variance across environments and point to possible connections to the intensity of selection on trait means.

## 1 INTRODUCTION

The micro‐environmental variance (V_e_) reflects the extent to which individuals living in a similar environment differ phenotypically due to small (“micro”) differences that they encounter within that environment (Falconer & Mackay, 1996; Lynch & Walsh, 1998). V_e_ is expected to be low in stable environments and increase as environments become more variable. In the laboratory, environmental conditions can be controlled and kept constant, minimizing chances that individuals experience micro‐environmental variation. Likewise, many animal and plant agricultural production units provide uniform environmental conditions in terms of factors such as temperature and access to similar food sources/nutrients throughout production life. In agroecosystems, environmental conditions are typically tuned to optimize production, but not necessarily fitness, and in such systems, uniformity in the product is typically economically advantageous. In evolutionary terms, this can be translated into an advantage of minimizing V_e_ (increasing uniformity within a defined environment) and potentially also the macro‐environmental variation (V_E_) occurring among defined environments such as different climatic regions (Hill & Mulder, 2010; Mulder, Bijma, & Hill, 2008).

There is limited information about mechanisms controlling sensitivity to micro‐environmental variation, but it appears to be trait‐specific and under some degree of genetic control (Hill & Mulder, 2010) and may therefore be influenced by selection (Bruijning, Metcalf, Jongejans, & Ayroles, 2020). When Hill and Mulder (2010) reviewed body weight and litter traits from (predominantly) mice, rabbits and livestock, they found evidence for heritable variation of V_e_ in most studies, but narrow sense heritabilities were low: in the range 0.00–0.05. More recently, a study on great tits (*Parus major*) provided evidence for genetic variance underlying V_e_ for fledging weight, likely maintained by selection for increased trait mean as it correlates with clutch size (Mulder, Gienapp, & Visser, 2016). Another selection experiment on the variance in litter size in rabbits also found evidence of heritable variation of V_e_ (Blasco, Martínez‐Álvaro, García, Ibáñez‐Escriche, & Argente, 2017). Studies on *Drosophila melanogaster* comparing genetically homogeneous lines or subpopulations have reported high broad‐sense heritabilities of micro‐environmental variance in four environmental stress tolerance traits (Morgante, Sørensen, Sorensen, Maltecca, & Mackay, 2015; Ørsted, Rohde, Hoffmann, Sørensen, & Kristensen, 2018; Sørensen, de los Campos, Morgante, Mackay, & Sorensen., 2015) and sleep traits (Harbison, McCoy, & Mackay, 2013). Overall, this suggests an evolutionary capacity to change the magnitude of phenotypic responses to a given micro‐environmental change, for example by increasing the micro‐environmental canalization to reduce V_e_ for fitness traits.

This emerging empirical evidence fits with the notion that V_e_ is exposed to and shaped by evolutionary forces (Bull, 1987; Gavrilets & Hastings, 1994; Slatkin & Lande, 1976), although the direction of evolutionary change expected in V_e_ is not entirely clear. Under relaxed selection on the mean, V_e_ is expected due to a cost of producing homogenous phenotypes (Lerner, 1954). Reviews of empirical selection gradients on the mean in natural populations have shown weak directional selection to be common (Blows & Brooks, 2003; Kingsolver, Diamond, Siepielski, & Carlson, 2012; Kingsolver et al., 2001). But many traits with a close connection to fitness are under strong stabilizing selection on the mean (for recent examples, see González Marín, Olave, Avila, Sites, & Morando, 2018; Sanjak, Sidorenko, Robinson, Thornton, & Visscher, 2018; Soulsbury, Siitari, & Lebigre, 2018). When stabilizing selection on the mean is strong and the trait mean approaches the optimum, genotypes increasing V_e_ will be at a disadvantage as they are likely to produce a nonoptimal phenotype (Bull, 1987; Gavrilets & Hastings, 1994; Slatkin & Lande, 1976). Hence, stabilizing selection on a trait's mean will cause correlated selection on V_e_, because V_e_ affects the probability of expressing an optimal trait value, and the closer to the optimal trait value the stronger the correlated selection for a reduced V_e_. Traits not directly linked to fitness may therefore have higher levels of V_e_. An increased V_e_ can also be the case for traits under strong directional selection, either as an indirect effect as the variance typically scales with a trait's mean (Falconer & Mackay, 1996), or as an direct effect because of selection for a higher mean (Hill & Zhang, 2004; Mulder, Bijma, & Hill, 2007). In the latter case, directional selection for an increased trait mean may also increase V_e_ scaled by the mean (CV_e_, coefficient of environmental variance) (Hill & Mulder, 2010).

When characterizing patterns of CV_e_ across environments, changes will reflect changes in V_e_ driven by environmental change or evolution, and not simply a correlated response to a change in trait mean. Hence, if a trait shows a reduction in CV_e_ in environments where selection on a trait's mean has been strong (a more optimal environment) compared to in environments where selection has been more relaxed (less optimal environment), we may expect this to be due to stabilizing or directional selection on a trait's mean. The expected magnitude of this reduction will depend on the environments compared, with a bigger reduction in CV_e_ when there is a large increase in optimality, and therefore a large increase in selection, from one environment to the other. However, strong directional selection might also lead to an increase in V_e_, because individuals with large V_e_ can produce the extreme phenotypes favoured by selection (Hill & Zhang, 2004; Mulder et al., 2007), whereby CV_e_ would show an increase in environments where selection on a trait's mean has been strong. These expectations also apply in the case when the environment of strong selection is heterogeneous, with the requirement that the same phenotype is optimal across environments (Gillespie & Turelli, 1989).

We have limited understanding of how selection aimed at increasing the trait mean affects V_e_ across environments, and if such selection can reduce the effects of environmental heterogeneity (Bruijning et al., 2020). This is partly because most quantitative genetic studies performed on natural populations only report estimates of heritability and only in very rare cases estimates of genotype–environment interactions for V_e_. When estimates of V_e_ are presented, they tend to have large confidence intervals due to low sample sizes. Estimates often lack clearly defined environmental contexts, limiting the ability to compare different sets of traits across studies. However, by focussing on livestock data, some of these problems are circumvented. Livestock studies are usually based on sample sizes an order of magnitude or more than those available from studies on natural populations (e.g., Carabano, Wade, & Vleck, 1990; Jamrozik, Schaeffer, & Weigel, 2002). Moreover, environments are typically highly controlled and well defined, and directly linked to mean values of production traits, limiting some of the complications that arise when considering selection in natural environments (Hunter, Pemberton, Pilkington, & Morrissey, 2018).

Here, we survey livestock literature on domestic cattle to examine patterns for V_e_ associated with different levels of selection and environmental variability. V_e_ can be approximated by residual variance (V_R_) estimates provided in the literature, but as the variance scales with the mean in most biological traits we here use the coefficient of residual variance (CV_R_, which is similar to CV_e_). Based on these data, we ask the following questions: (a) Does environmental heterogeneity increase trait CV_R_ or decrease it on the assumption that strong stabilizing selection on trait means buffer phenotypic heterogeneity in the environment? (b) Is there a consistent difference in CV_R_ in more optimal environments compared to less optimal environments for traits that are expected to have been under past stabilizing versus directional selection? and (c) Is there an association between CV_R_ and CV_E_ (V_E_ scaled by the trait mean) across traits, as might be expected if environmental variance components for different traits are influenced by the same processes (Debat & David, 2001)?

## 2 MATERIALS AND METHODS

### 2.1 The survey

In the livestock literature, studies of genotype by environment interactions typically consist of quantitative genetic analyses of traits across several well‐defined environments, providing useful estimates of V_e_ (i.e., V_R_) from each of these environments. Genotype‐by‐environment interactions are often assessed using the genetic correlation of a trait between two environments (Falconer, 1990). We undertook a literature survey with the search terms “environments” AND “genetic correlation*” AND “cattle” (assessed: 15/09/17) on the Web of Science Core Selection (http://www.webofknowledge.com). To identify useful papers with high‐quality data, we set up the following strict criteria for a study to be added to our database: (a) the study comprises at least two different environments enabling direct comparison within study system; (b) the environments are clearly defined by geography, abiotic conditions or rearing conditions, allowing an assessment of the level of environmental quality (see below); (c) the study provides V_R_ or, if V_R_ is not provided, parameters necessary for estimation of V_R_ (h^2^, V_A_, V_P_); and (d) the paper provides mean trait values for each environment, allowing scaling of V_e_. Studies where environments were solely defined by the grouping of herds according to differences in mean trait values of another trait than that of interest (typically milk yield is used) were not considered. The reference lists of the identified papers were used to identify additional papers complying with the above requirements. When multiple papers presented similar analyses on the same dataset, we only included data from the first paper identified. Only papers written in English were included. These criteria reduced the number of papers from 377 to 33 papers containing 305 data points, and the large reduction in number of papers was mostly due to missing data (criteria 3 and 4) or because comparisons were between traits or within traits between different time‐points (i.e., weaning, entry, parity, lactation, days in milk) instead of between environments. This literature search is based on strict criteria to ensure high‐quality data and is not meant to be exhaustive. The literature search and several elements of data extraction as well as trait definitions described in the current study (and the description thereof) are shared with another study where we investigate the dependence of genetic correlations on environmental similarity (Schou, Hoffmann, & Kristensen, 2019).

From each study, we extracted trait mean and V_R_ or parameters necessary for the estimation of V_R_ (h^2^, V_A_, V_P_). We also extracted repeatability when available or estimated repeatability based on estimates of V_e‐perm_ (the permanent environmental variance: the phenotypic variance among individuals which is permanent across multiple measurements) if this parameter was available. When estimates were only graphically available, we extracted numerical data using WebPlotDigitizer (://arohatgi.info/WebPlotDigitizer/app/). Some of the studies that passed the above criteria used random regression analysis to estimate genetic correlations across a defined environmental dimension (e.g., ambient temperature in Ravagnolo, Misztal, & Hoogenboom, 2000). For these studies, we extracted estimates of trait mean and V_R_ from the most extreme environments (with a suitable sample size) and from an intermediate environment, according to the trait mean.

### 2.2 Optimal environments

Environments in studies on domestic animals are typically defined by traits related to production. Environments are assumed more optimal when high milk yield or weight gain in cattle is observed. High trait values might also reflect genetic improvement although such effects are minimized by focussing on one breed or else controlling for breed effects. We therefore use trait mean as a measure of proximity to the optimal environment (optimality); that is, the most optimal environment for the trait of interest is the one giving the highest mean, and therefore, the environment targeted in the selection process.

As an alternative to describing an environment using trait mean, we also classified each environment into one of three levels of environmental quality (similarity to the environment targeted in the selection process) using country of rearing and environmental description provided in the study. We grouped countries according to latitude and level of development (Table S1). We acknowledge that this grouping is artificial, but it is nevertheless useful as it disconnects trait mean from the inferred environmental optimality and thereby circumvents potential issues with genetic improvement affecting trait mean. Canada and countries from North‐Western Europe were assumed to have highly technical and modern rearing conditions as well as high levels of animal welfare, resulting in high levels of environmental quality. Countries from Eastern and Mediterranean Europe as well as highly developed countries in the Americas (excluding Canada) and Africa including South Africa were assumed to have somewhat lower levels of quality and increased levels of grazing. These were therefore classified as having intermediate environmental quality. The remaining countries from the Americas and Africa as well as Middle Eastern countries were classified as having a low environmental quality. However, if a reference provided environmental details indicating lower quality, such as lack of environmental control due to grazing, we subsequently decreased the level of quality independent of location (Table S1). Conversely, if environments were described as being particularly high quality, for example, by intensive management or feedlot rearing, we incremented the level of quality again independent of location. Based on this classification, environments with low quality are also expected to be less optimal as measured by trait means, and this approach therefore provides a different way to rank environments. The two measures of optimality were compared in studies where traits of the same breeds were measured at several levels of quality (Figure S1). Across studies and traits, optimality measured by trait mean was higher in environments of intermediate than of low quality (Wilcoxon signed‐rank test: *V* = 1,092, *p* < .001) and higher in environments of high compared to intermediate quality (Wilcoxon signed‐rank test: *V* = 1, *p* = .016).

### 2.3 Trait definitions

We excluded traits under direct farmer control such as age of culling, and traits that could not be related to a distinct biological trait such as lifetime net income (see Table S2 for trait definitions). If a paper provided data on milk yield in several lactations, we used data from the first lactation, as this was the most prevalent across studies, and reduced issues associated with culling of low production individuals (Banos & Shook, 1990). Trait estimates related to milk production were typically reported on Holstein cattle (denoted Holstein–Friesian, Friesian or Holstein), while trait estimates related to beef production were mostly reported from Angus and Hereford or various mixed breeds (Figure S2). To investigate how selection has affected V_R_ across traits having some shared evolutionary history, we grouped traits into five categories based on evolutionary principles: disease indicator, growth, life history, morphology and physiology (Hoffmann, Merilä, & Kristensen, 2016; Roff & Mousseau, 1987) (Table S2). The trait category disease indicator was omitted from all analyses as we only obtained four estimates of V_R._

### 2.4 Statistical analyses

#### 2.4.1 Estimating differences in V_e_ between environments (general approach)

To enable comparisons across and within traits, we scaled the variance by the mean by calculating CV_R_ (coefficient of residual variance that includes all terms except V_A_), which is therefore used as a proxy of V_e_ throughout the analyses. For each trait in a study, we estimated the difference in CV_R_ between two environments by the coefficient of variation ratio (lnCV_R_) (Morrissey, 2016; equation 11 in Nakagawa et al., 2015):

$$\ln{\text{CV}}_{R\;\text{optimal}}=\ln\left(\frac{{\text{CV}}_{R\;\text{more}\;\text{optimal}}}{{\text{CV}}_{R\;\text{less}\;\text{optimal}}}\right)+\frac{1}{2(n_{\text{more}\;\text{optimal}\;-\;\text{1}})}-\frac{1}{2(n_{\text{less}\;\text{optimal}-1})}$$

Here, a negative lnCV_R optimality_ reflects a decrease in CV_R_ in the more optimal environment, while a positive lnCV_R optimality_ reflects an increase in CV_R_ in the more optimal environment. We considered the environment with the highest trait value of each pair of environments as the more optimal (see above). The first term in the formula is the natural logarithm of CV_R_ in the two environments, while the two additional terms are used to control for differences in sample size (i.e., the number of individuals). When the number of individuals was not provided in the reference, we used the number of records. The sampling variance of lnCV_R_ was estimated using equation 12 in Nakagawa et al. (2015), which also relies on the sample size. This equation was not derived with the uncertainty of the estimated variances from animal models in mind and may therefore underestimate this variance estimate; however, this is negligible because of the large sample size. The use of lnCV_R_ relies on the assumption that SD_R_ and trait mean are linearly correlated, which has been verified for this dataset (Figure S3). We estimated lnCV_R optimality_ for all possible pairs of CV_R_ estimates within each trait in a study, and only within breeds. This meta‐analytic approach incorporates sample size and circumvents several issues with the use of ratios (CV_R_ is a ratio) in statistical analyses. Comparing CV_R_ among traits of different dimensionalities can yield spurious differences as higher dimensionality causes higher CV_R_ (Houle, 1992). However, this is not a problem in our dataset as no 3D traits are included in the survey.

#### 2.4.2 Comparing CV_R_ between heterogeneous and controlled environments

Eight studies reported estimates of CV_R_ from two contrasting environments, one more heterogeneous (*organic* or *grazing*) and the other more homogeneous (*conventional*, *confinement*, *total mixed ration* or *feedlot*). We compared CV_R_ between these environments by estimating lnCV_R homogeneity_.

$$\ln{\text{CV}}_{R\;\text{homogeneity}}=\ln\left(\frac{{\text{CV}}_{R\;\text{homogeneous}}}{{\text{CV}}_{R\;\text{hetrogeneous}}}\right)+\frac{1}{2(n_{\text{homogeneous}-1})}-\frac{1}{2(n_{\text{hetrogeneous}-1})}$$

Here, a negative lnCV_R homogeneity_ reflects a decrease in CV_R_ in the homogeneous environment, while a positive lnCV_R homogeneity_ reflects an increase in CV_R_ in the homogeneous environment. For this particular analysis, a limited number of estimates of lnCV_R_ were available (*n*_Growth_ = 7, *n*_Life history_ = 9, *n*_Morphology_ = 15 and *n*_Physiology_ = 20), preventing usage of generalized linear mixed models (GLMMs) accounting for study, trait and breed, and we therefore undertook one‐sample *t* tests to assess whether the lnCV_R_ of each trait category differed from zero.

### 2.5 Testing the dependence of CV_R optimality_ on the distance to trait optimum

To investigate the change in CV_R_ with increasing proximity to a trait optimum, we tested if the difference in trait mean between two environments correlated with their difference in CV_R_ (lnCV_R optimality_). The difference in trait mean ($\bar{x}$) was estimated as the response ratio (lnRR), with the highest trait value being defined as closer to the optimum and therefore more optimal.

$$\ln\text{RR}=\ln\left(\frac{{\bar{x}}_{\text{more}\;\text{optimal}}}{{\bar{x}}_{\text{less}\;\text{optimal}}}\right)$$

As the numerator is by definition bigger than the denominator, lnRR will always be positive, and a higher lnRR will reflect a higher increase in optimality from one environment to the other.

We tested the effect of lnRR on lnCV_R optimality_ in each of the trait categories using a linear mixed model (Gaussian distribution) in a Bayesian framework in the R‐package MCMCglmm (v.2.25) (Hadfield, 2010). We specified lnRR (continuous) and trait category (factorial) as well as their interaction as the fixed effects. Study, trait and breed were specified as random effects, with random slopes (unstructured covariance–variance matrix) of breed across lnRR. We accounted for the estimated sampling variance by using the build‐in argument *mev* that is specifically designed to handle sampling variance. We specified the random effects and residual priors as (uninformative) inverse gamma priors (*V* = 1, *nu* = 0.002) and performed 1,000,000 iterations of which the initial 20,000 were discarded and only one in 500 runs was used for estimating posterior probabilities. Convergence of the estimates was checked by running the model four times and inspecting trace plots and their overlap of the MCMC chain and the level of autocorrelation among posterior samples.

Within each trait category, some traits may show trait‐specific responses to lnRR. Our literature survey resulted in 368 estimates of lnCV_R optimality_, but with a highly skewed distribution across a high number of traits (*n* = 52). 43 traits had less than seven estimates and only four traits had more than 15 estimates. As a consequence, we focused on the four traits with highest replication: yearly milk yield *(305‐d milk yield*), birthweight, bodyweight at weaning (*200–210‐d body weight*) and dry matter intake. To analyse the effect of lnRR on lnCV_R optimality_ in each of these traits, we followed the same approach as for the trait category analysis (priors, iterations, sampling and convergence check). We specified lnRR as the sole fixed effect and study as a random effect. As three of the traits predominantly consisted of estimates from one breed (yearly milk yield: *n*_Holstein_ = 37, *n*_Guernsey_ = 6, *n*_Jersey_ = 1; birthweight: *n*_Angus_ = 37, *n*_Hereford_ = 3; dry matter mass: *n*_Holstein_ = 28) and the last trait from two breeds (bodyweight at weaning: *n*_Angus_ = 38, *n*_Hereford_ = 21; *n*_MixedTemperateMeat_ = 3), we restricted the analysis to these breeds.

### 2.6 Comparing CV_R_ between environments of different quality

As an alternative to using trait mean as a measure of how optimal an environment is in terms of proximity to trait optimum, we investigated whether our classification into environmental quality was related to estimates of CV_R_ in each of the trait categories. We used the meta‐analytic framework from above and compared CV_R_ of a trait measured at two different environmental qualities within a study using lnCV_R quality_.

$$\ln{\text{CV}}_{R\;\text{quality}}=\ln\left(\frac{{\text{CV}}_{R\;\text{higher}\;\text{quality}}}{{\text{CV}}_{R\;\text{lower}\;\text{quality}}}\right)+\frac{1}{2(n_{\text{higher}\;\text{quality}}-1)}-\frac{1}{2(n_{\text{lower}\;\text{quality}}-1)}$$

Here, a negative lnCV_R quality_ reflects a decrease in CV_R_ in the higher quality environment, while a positive lnCV_R quality_ reflects an increase in CV_R_ in the higher quality environment. To test whether the lnCV_R quality_ of the environmental contrasts was predominantly below or above zero, we used a linear mixed model with the same modelling approach as for lnRR (priors, iterations, sampling and convergence check). We specified environmental contrast (High vs. Intermediate, High vs. Low or Intermediate vs. Low) and trait category as well as their interaction as fixed effects. Study, trait and breed were specified as random effects.

### 2.7 Comparing V_e_ and V_E_

If micro‐environmental change has the same phenotypic effects as macro‐environmental change, a trait showing high V_E_ is predicted to also show high V_e_ and *vice versa*, resulting in a linear association between V_e_ and V_E_ across traits. To estimate V_E_, we need measurements at multiple well‐defined environments within a study, but a comparison across traits and studies is only possible if the same environments are tested for all traits, excluding the majority of the surveyed literature. The largest subset of our surveyed literature fulfilling these requirements was three studies encompassing a total of 17 traits, all measured in two different feedlot environments and one pasture‐based environment (Johnston, Reverter, Burrow, Oddy, & Robinson, 2003; Johnston, Reverter, Ferguson, Thompson, & Burrow, 2003; Reverter, Johnston, Perry, Goddard, & Burrow, 2003). We estimated V_E_ as the variance of trait means in the three environments and standardized this measure by the trait mean across environments (CV_E_). Hence, for this analysis the data structure is one estimate of CV_E_ and multiple estimates of CV_R_ (one for each of the three environments) for each trait in each study, preventing usage of lnCV_R_ as a measure of difference in variability. We therefore investigated the relationship between CV_R_ and CV_E_ by fitting a linear mixed model with CV_R_ as the response variable and CV_E_ as the sole fixed effect. Study and trait were specified as random effects, whereas breed was not included as all data originated from one breed. The model was fitted using MCMCglmm (v.2.25) (Hadfield, 2010) using a similar modelling approach (priors, iterations, sampling and convergence check) as for the lnRR analysis.

## 3 RESULTS

### 3.1 Does the heterogeneity of environments affect CV_R_?

**Figure 1:**
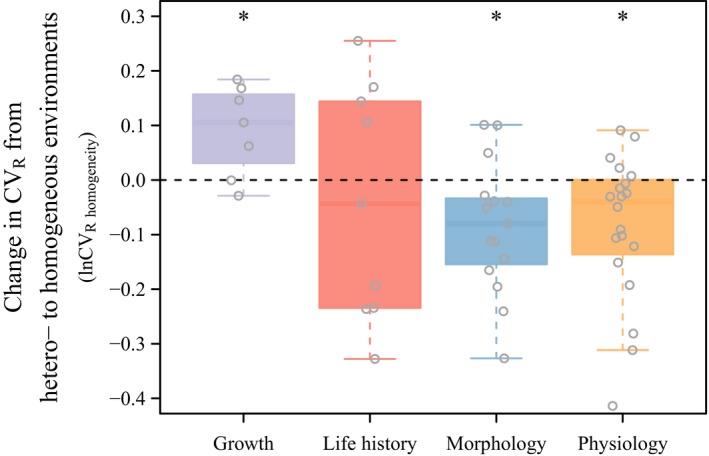
Boxplot comparing CV_R_ between heterogeneous and homogeneous environments. Eight studies compared the performance of cattle in environments contrasted by their difference in rearing, where one was characterized as more heterogeneous (free‐ranging, including organic or grazing) and the other environment as more homogenous and controlled (conventional, confinement, total mixed ration or feedlot). CV_R_ is a proxy of V_e_ and was estimated as the coefficient of residual variance that includes all terms except V_A_. To compare the CV_R_ between the two environments within a study, we calculated the coefficient of variation ratio (lnCV_R homogeneity_). lnCV_R homogeneity_ measures the relative change in CV_R_ from a heterogenous to a more homogeneous environment, and hence, negative values indicate lower CV_R_ in the more homogenous environment and vice versa. Trait‐specific estimates are presented in Figure S4. *p < .05

When comparing CV_R_ in heterogeneous environments (i.e., free‐ranging) and homogeneous environments (i.e., confined) (Figure S4 for trait‐specific data), the production‐related trait category of growth traits showed a higher CV_R_ in homogenous environments compared to heterogeneous environments (a positive lnCV_R homogeneity_: *t*_(6)_ = 2.93, *p* = .027; Figure 1). The other production‐related trait category consisting of life history traits showed no difference between the two environments (*t*_(8)_ = −0.55, *p* = .592). However, morphological traits (a negative lnCV_R homogeneity_: *t*_(14)_ = −2.76, *p* = .015) and physiological traits (a negative lnCV_R homogeneity_: *t*_(19)_ = −2.85, *p* = .010) both had a lower CV_R_ in the homogenous than in the heterogeneous environment. It should be noted here that low levels of replication made us prefer simple one‐sample *t* tests and not the more robust meta‐analytic approach used for the other analyses, and the results should be interpreted with this mind.

### 3.2 Does CV_R_ decrease in more optimal environments?

**Figure 2:**
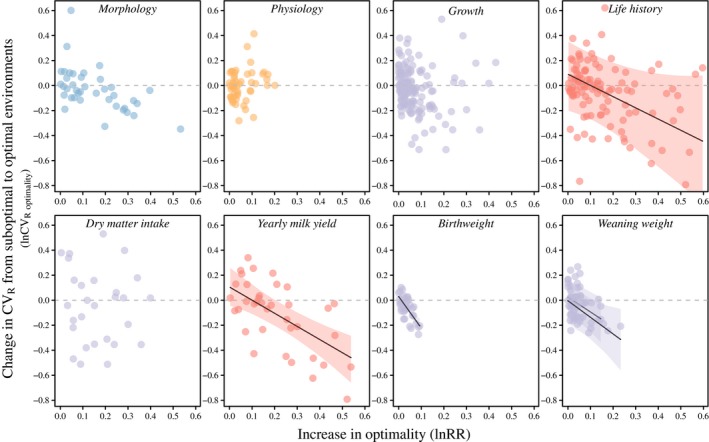
Association between CV_R_ and proximity to trait optimum (optimality). CV_R_ is a proxy of V_e_ and was estimated as the coefficient of residual variance that includes all terms except V_A_. We used trait mean as a measure of proximity to trait optimum (higher trait mean being more optimal) and measured the relative change in CV_R_ from a suboptimal to a more optimal environment using lnCV_R optimality_. We then tested if lnCV_R optimality_ was affected by the difference in optimality between the two environments (lnRR). In traits from the life history category, we found a greater reduction in CV_R_ (negative lnCV_R optimality_) to correlate with the increase in optimality (increased lnRR). This was supported by a similar correlation in the production‐related trait yearly milk yield (Holstein cattle). No relationship was found for the other trait categories; however, the production‐related growth traits birthweight (Angus cattle) and weaning weight (upper line: Angus and lower line: Hereford) also showed a reduction in CV_R_ with increasing optimality of the rearing environment (Table 1). Colours illustrate the four trait categories: morphology (blue), physiology (yellow), growth (purple) and life history (red). The shaded area around the fitted line represents the 95% confidence interval

When testing the change in CV_R_ from suboptimal to more optimal environments (lnCV_R optimality_), one prediction is that no or a very small increase in optimality (lnRR ~ 0) should result in no or little change in CV_R_ (lnCV_R optimality_ ~ 0). This prediction was confirmed across trait categories and traits (Figure 2) with intercepts estimated to be close to zero (Table 1). We hypothesized that as the increase in optimality becomes larger (lnRR » 0), CV_R_ should decrease due to historical selection in these environments, represented by a negative relationship between lnRR and lnCV_R optimality_. This prediction was confirmed for life history traits, whereas no significant effects were identified for the other trait categories (Figure 2, Table 1). The decrease in lnCV_R optimality_ was also found when focusing on the life history trait yearly milk yield (Figure 2, Table 1). Traits in the growth trait category are all traits measuring body weight at a certain age, except the trait dry matter intake which is a measure of feed intake. Both body weight at weaning and birthweight showed a decrease in CV_R_ when measured in more optimal environments, but there was no effect on dry matter intake (Figure 2, Table 1). In line with this, a separate analysis of the growth category without dry matter intake resulted in a significant decrease in lnCV_R optimality_ with increasing difference in optimality (lnRR = −1.55; CI = −3.83 to −0.04; *p*_MCMC_ = .036).

**Table 1:** Testing the change in CV_R_ from suboptimal to more optimal environments using GLMMs in MCMCglmm

Model	Fixed effect	Posterior mean (CI)	*p*_MCMC_
Trait categories	Growth	0.02 (−0.24;0.28)	.836
	Growth: lnRR	−0.67 (−1.44;0.13)	.075
	Physiology	−0.06 (−0.33;0.22)	.643
	Physiology: lnRR	1.08 (−0.25;2.45)	.112
	Morphology	0.06 (−0.19;0.35)	.643
	Morphology: lnRR	−0.37 (−1.34;0.77)	.444
	Life history	0.09 (−0.19, 0.37)	.472
	Life history: lnRR	−0.91 (−1.76;−0.08)	**.021**
Dry matter intake	Intercept	−0.02 (−0.23;0.19)	.836
	lnRR	−0.17 (−1.20;0.93)	.746
Body weight at weaning	Breed (Angus)	0.03 (−0.12;0.16)	.599
	Breed (Angus): lnRR	−1.15 (−2.01;−0.28)	**.019**
	Breed (Hereford)	0.00 (−0.20;0.15)	.905
	Breed (Hereford): lnRR	−1.33 (−2.64;0.17)	**.037**
Birthweight	Intercept	0.06 (−0.39;0.42)	.943
	lnRR	−2.23 (−3.25;−1.22)	**<.001**
Milk yield	Intercept	0.14 (−0.17;0.46)	.228
	lnRR	−0.94 (−1.36;−0.51)	**<.001**

Note

A term was considered statistically significant when the *p*_MCMC_ value < .05 marked in bold.

The change in CV_R_ (a proxy of V_e_) from suboptimal to more optimal environments was measured by lnCV_R optimality_, while the relative difference in optimality between two environments (always from suboptimal to optimal) was measured by lnRR. Hence, a negative lnCV_R optimality_ reflects a decrease in CV_R_ in the more optimal environment. We tested the effect of lnRR on lnCV_R optimality_ in a model with the four trait categories (growth, physiology, morphology and life history) and in trait‐specific models for the four traits with *n*_estimates_ > 15. In these models, a negative relationship of lnRR on lnCV_R optimality_ reflects that a greater reduction CV_R_ (negative lnCV_R optimality_) occurs when the increase in optimality is large (increased lnRR). The models were constructed such that the regression lines and intercepts of each trait category/trait were compared to an lnCV_R optimality_ of zero.

**Figure 3:**
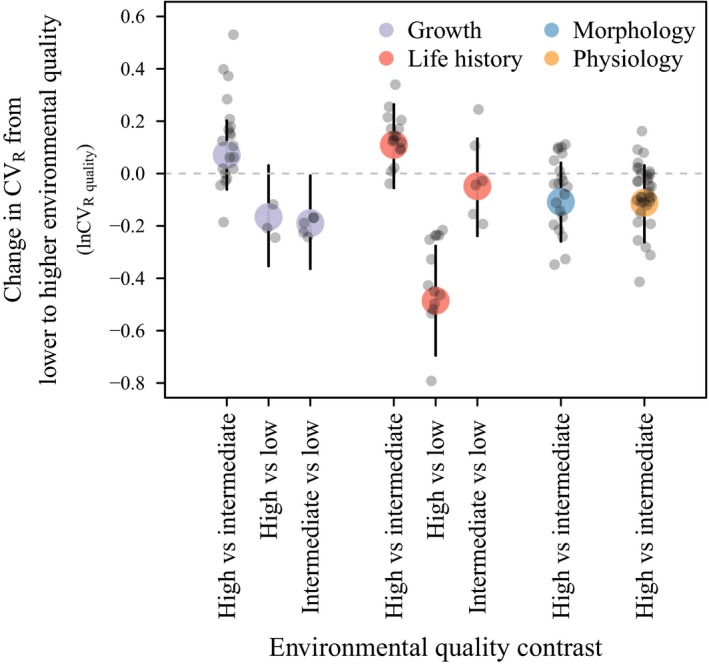
Change in CV_R_ from lower to higher environmental quality. Environmental quality (a measure of optimality) was assigned to each CV_R_ estimate using information on country of origin and environmental details from the reference. CV_R_ is a proxy of V_e_ and was estimated as the coefficient of residual variance that includes all terms except V_A_. The change in CV_R_ from lower to higher environmental quality was measured by lnCV_R quality_. Several of the environmental quality contrasts of growth and life history traits showed a decrease in CV_R_ in environments of higher quality (a negative lnCV_R quality_). Points and error bars show the posterior mean and 95% confidence intervals estimated in GLMMs in MCMCglmm

We also tested the impact of environmental quality on CV_R_, as an alternative to the use of mean trait value as a measure of environmental optimality. Our assessment of quality was based on both geographical and environmental information provided in the studies, and resulted in 117 estimates of lnCV_R quality_ comparing different qualities. When we considered life history traits (Figure S5 for trait‐specific CV_R_ estimates), we found a significantly lower CV_R_ at high compared to low environmental quality, represented by a negative lnCV_R quality_ (High vs. Low = −0.49; CI = −0.71 to −0.27; *p*_MCMC_ < .001; Figure 3, Table S3). This was also the case when comparing CV_R_ of growth traits between environments of intermediate and low quality (Intermediate vs. Low = −0.19; CI = −0.38 to −0.01; *p*_MCMC_ = .048; Figure 3, Table S3). None of the environmental contrasts were significant for the physiological or morphological trait categories (Figure 3, Table S3).

### 3.3 Is there an association between CV_R_ and CV_E_ across traits?

**Figure 4:**
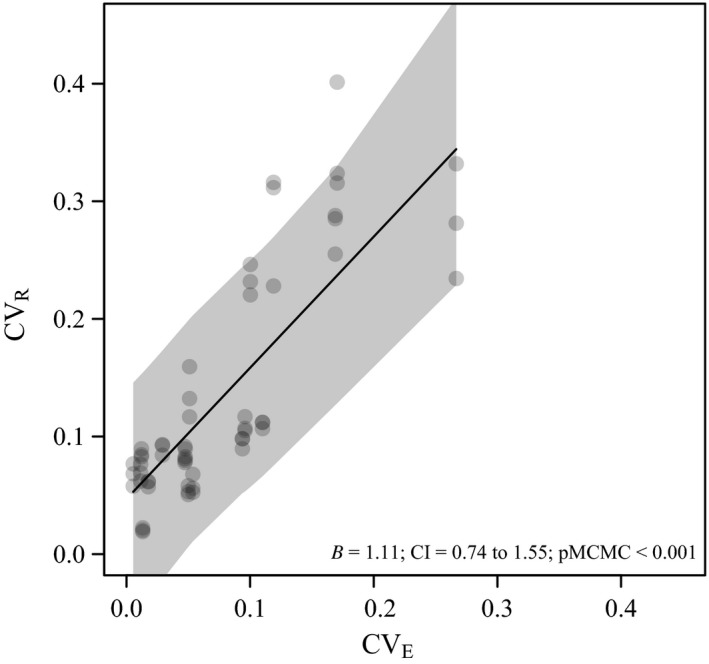
Association between CV_E_ and CV_R_. Meaningful comparisons of phenotypic variance among and within environments require that all traits are measured in the same combination of environments. Three of the studies in our literature survey measured performance across 17 traits, and all measured in two different feedlot environments and one pasture‐based environment (Johnston, Reverter, Burrow, et al., 2003; Johnston, Reverter, Ferguson, et al., 2003; Reverter et al., 2003). Environmental variance across environments (V_E_) was estimated as the variance in trait means in the three environments. We tested the effect of CV_E_ on CV_R_ using a linear mixed model. The shaded grey area around the fitted line represents the 95% confidence interval

In the data extracted from three studies where all traits were measured in the same three environments, we found the traits with high within environmental variance (CV_R_) to also have significantly higher among environmental variance (CV_E_) (Figure 4).

### 3.4 Patterns of CV_R_ across traits

From the literature survey, we obtained 306 estimates of CV_R_ (Table S4), which are grouped by traits and presented in a boxplot (Figure S6), along with within‐individual environmental variance (CV_WI_) estimated from repeatability and V_P_. Traits in the growth category had particularly low CV_R_ with the exception of two traits measuring growth in narrow time intervals (weight gain and daily weight gain). The same pattern was observed for production traits related to life history, where traits measured across long time intervals (305 days) showed relatively low CV_R_ in comparison with the limited time intervals of daily yield. The life history traits related to reproduction spanned the entire range of CV_R_, with age at first calving and gestation period being as low as the production traits, while traits quantifying the time interval between two reproduction events had higher CV_R_. Survival is dependent on farmer culling, and this trait also had a very high estimate of CV_R_. Morphological traits were distributed in two groups, one consisting of classical morphological traits such of size of a given muscle, which had low CV_R_, and a second describing the amount of fat in the muscles which had high CV_R_. The physiological traits comprised both the per cent fat and protein of milk, physiological characteristics of muscles as well as rectal temperature, which all generally had low CV_R_.

## 4 DISCUSSION

### 4.1 CV_R_ in heterogeneous environments

Adaptive evolution has long been thought to be involved in shaping phenotypic sensitivity to micro‐environmental variance (Van Valen, 1962; Waddington, 1942). Here, we used estimates of V_e_ from the domestic cattle literature (estimated as CV_R_) to identify patterns of V_e_ across environments with different histories of selection and different micro‐environmental variation. As expected, we found CV_R_ to increase when a cattle breed is reared in heterogeneous environments and spends more time in pastures, compared to when the cattle breed is reared in more controlled environments predominantly involving confinement (Figure 1). This fits well with the expectations for traits under relaxed selection, where a more heterogeneous environment is predicted to increase V_e_ due to increased chances of encountering different conditions each having distinct effects on the phenotype. However, this was not the case for traits linked to production (growth and life history traits). Here, environmental heterogeneity decreased CV_R_ (growth) or had no effect on CV_R_ (life history). Production traits are expected to be under the strongest selection towards a constant trait optimum across environments. Such stabilizing selection can potentially reduce or remove any association between heterogeneity and V_e_ (Gillespie & Turelli, 1989), resulting in evolution of a decreased influence of environmental heterogeneity on V_e_ in these traits. The observed signature of selection in domestic cattle is unlikely to exist for similar traits in natural populations (Kingsolver et al., 2001) living in more heterogeneous environments. For instance, natural populations are likely to face fluctuating food availability and large variation in abiotic factors such as temperature and variation, which are reduced in livestock in confinement being fed ad libitum. However, in natural populations other traits such as those related to species recognition would be expected to be under strong stabilizing selection favouring the same optimal phenotype across environments.

### 4.2 Selection for decreased CVR in more optimal environments

When using an increased trait value as a proxy for a more optimal environment, we found evidence for a decrease in CV_R_ in more optimal environments in life history and growth traits. This may reflect stabilizing selection; when the trait mean approaches the optimum trait value (or the optimum environment), high V_e_ becomes disadvantageous and a reduction in V_e_ may be expected under stabilizing and directional selection (Bull, 1987; Gavrilets & Hastings, 1994; Slatkin & Lande, 1976). On the other hand, strong directional selection may increase V_e_ as extreme phenotypes are favoured by selection (Hill & Zhang, 2004; Mulder et al., 2007), but this is not supported by the pattern we observed. Our findings suggest that strong selection on the mean trait value under optimal rearing conditions favours a reduced micro‐environmental variance for these traits (Figure 2). Indeed, the life history and growth traits are typically associated with milk production (e.g., yearly fat yield and yearly milk yield) and beef production (weaning weight and birthweight); traits that have all been under strong selection. It is however possible that optimal environments are also more homogenous, so that lower micro‐environmental variation and not environmental optimality are driving lower CV_R_. As we found an increase in CV_R_ of growth traits in more homogenous environments (Figure 1) and evidence for a reduction in CV_R_ in more optimal environments (Figure 2), we suspect that selection on V_e_ is involved.

We also considered another measure of environmental optimality which disregarded trait mean and instead assigned each CV_R_ to three quality levels (environmental quality). This approach puts the environment on one single scale across traits, allowing us to test for overall differences in each trait category. The grouping of environments was based on the grounds that the vast majority of estimates of CV_R_ used in this study originate from Holstein, Angus and Hereford cattle (or varieties hereof), all originally Northern European breeds (Decker et al., 2014) and still bred for performance in highly controlled environments. For both growth and life history traits, our results supported a decrease in CV_R_ in environments classified as having a higher quality (Figure 3). This was not significant for all contrasts, but suggests some support for the findings obtained when using trait value as a proxy for a more optimal environment. The lower selection intensity and more variable nature of selection and trait‐fitness associations in natural populations (Hunter et al., 2018) would make it much harder to detect such a signal in wild populations. Recently, a meta‐analysis based predominantly on laboratory studies of fish and insects presented support for a reduced residual variance in benign compared to stressful environments (Rowiński & Rogell, 2017).

### 4.3 Patterns of CV_R_ across traits

Estimates of CV_R_ were relatively low for growth traits (Figure S6) likely to have been under strong directional selection during domestication (Rauw, Johnson, Gomez‐Raya, & Dekkers, 2017) as well as for a subset of the morphological traits important for beef production. In comparison, traits related to storage of energy surplus, both intra‐ and extra‐muscular fat (P8 on Figure S6) had high estimates of CV_R_. The phenotypic optimum of these traits may have varied in the past given that optimal levels of fat storage are expected to vary across seasons and years due to changing weather. Such fluctuating selection due to environmental heterogeneity has also been observed for male horn growth in a wild population of Soay sheep (Robinson, Pilkington, Clutton‐Brock, Pemberton, & Kruuk, 2008) and is an example of how higher levels of V_e_ can be maintained by selection in natural populations. Patterns for life history traits fell into two groups. One group had a high CV_R_, consisting of milk production traits measured over short time scale that likely have low repeatability (high CV_WI_) increasing the lower limit of CV_R_. This group also included reproduction traits quantifying the time interval between two reproduction events that will vary between seasons and years. The other group of traits with lower CV_R_s included yearly milk production and measures of gestation period and thus milk production, both traits expected to have been directionally selected under and after domestication. While these traits showed low micro‐environmental sensitivity compared to the other life history traits, CV_R_ of milk production traits was surprisingly high given their link to production. Perhaps CV_R_ is constrained by a low repeatability, but care should be taken here, as repeatability is measured across lactations, which may represent partly distinct traits (Haile‐Mariam & Pryce, 2015; Yamazaki, Takeda, Osawa, Yamaguchi, & Hagiya, 2019).

Relating these patterns to those measured in natural populations is challenged by the scarcity of studies on this subject. Some of the closest relatives to the domesticated cattle include the highly domesticated water buffalo, while nondomesticated species (or to a lesser extent) include the common eland, bison species, the African buffalo, the rare saola, the addax as well as wildebeest and gazelle species (Bibi, 2013). Few genetic analyses have been performed on these species, with the exception of a study on the highly inbred Cuvier's gazelle (Ibáñez, Cervantes, Gutiérrez, Goyache, & Moreno, 2014) in which estimates on the environmental variance are not provided. The only study we could find comparing CV_R_ across traits in a wild mammal population (red deer) also found higher CV_R_ in life history traits than for morphological traits (leg length and jaw length) and birthweight (Kruuk et al., 2000). However, measured life history traits were mostly fitness traits influenced by longevity, which is a complex phenotype that is also influenced by stochastic events.

### 4.4 An association between CV_R_ and CV_E_ across traits

Macro‐environmental variance (V_E_) is typically presented as reflecting large changes in a few controlled environmental parameters, while V_e_ reflects variation in an infinite number of environmental parameters. However, there may still be overlap in genetic factors contributing to V_e_ and V_E_, which leads to the prediction that traits with low sensitivity to V_e_ will have a relatively low sensitivity to V_E_. We found support for this relationship (Figure 4), but because such comparisons are only permissible when the same micro‐ and macro‐environments are assessed across traits, the trait subset was rather restricted, which precludes comparisons among trait groups. In *Drosophila melanogaster*, partitioning of four different components of the environmental variance of cold tolerance revealed significant genetic correlations among these components (Ørsted et al., 2018). Although only performed on one trait, it suggests that components of environmental variation can be partly described by a common axis of environmental change (Debat & David, 2001). We speculate that in natural populations, traits under selection for a buffering of macro‐environmental changes, will show a decreased micro‐environmental sensitivity, and *vice versa*. Such buffering can be described as canalization of phenotypic perturbations, for example, by the expression of epistatic genes, a mechanism known to evolve and buffer the influence of both small‐ and large‐scale environmental variation across multiple environmental parameters (Flatt, 2005).

### 4.5 Livestock literature in evolutionary biology

Drawing general conclusions on the evolution of environmental sensitivity is often limited by restrictions on sample size and experimental constraints when investigating natural and laboratory populations. To overcome these issues, we have exploited the extensive livestock literature. However, we acknowledge that this approach is based on several assumptions and the relevance of the conclusions to natural populations remains to be tested.

In order to make V_e_ (approximated by V_R_) comparable across environments and studies, we followed typical practice and standardized V_e_ by the mean (Kruuk et al., 2000; Rowiński & Rogell, 2017). This relies on the assumption that the variance scales with the mean in biological traits and that selection for increased mean causes a correlated response in the variance (Falconer & Mackay, 1996); hence, we assume that deviations from this relationship therefore reflect evolutionary change in V_e_. This assumption is supported by positive variance–mean relationships in most of the tested traits (Figure S3).

When interpreting differences in CV_R_ across environments, we assume a history of directional and/or stabilizing selection on a trait's mean, of varying strength across traits. However, the distinction between the two modes of selection may not be clear in cattle breeds. Mean levels of most production‐related traits (milk and meat) have increased across the last century through strong directional selection aided by progeny testing (Robertson & Rendel, 1950) where superior bulls are selected on the basis of the performance of their offspring in a multiple trait index (Hazel, 1943). But due to morphological and/or physiological constraints, there are likely to be negative fitness consequences of very high production outputs (Rauw, Kanis, Noordhuizen Stassen, & Grommers, 1998). Therefore, an upper limit has likely been indirectly enforced during selection on these traits, resulting in some form of stabilizing selection (García‐Ballesteros, Gutiérrez, Varona, & Fernández, 2017)*.* Cattle were domesticated approximately 10,500 years ago (Bollongino et al., 2012; Scheu et al., 2015). Following this, processes of artificial selection were likely practised for thousands of years before selection became based on modern multi‐trait progeny testing procedures. Our interpretations are therefore based on the assumption that traits have evolved substantially since domestication, also before modern selection programs were used, and that the current trait values have been shaped by some form of stabilizing selection.

The finding of a low CV_R_ in environments with high trait values and a high CV_R_ in environments with low trait values was interpreted to be a product of selection. This relies on the assumption that environments where high trait values are observed are more optimal and on the assumption that the most optimal environments are the environments where selection for production traits occurs in domestic cattle. In such a system, evolutionary theory predicts that if stabilizing selection on the mean is strong and the trait mean approaches the optimum, genotypes increasing V_e_ will be at a disadvantage as they are likely to produce a nonoptimal phenotype (Bull, 1987; Gavrilets & Hastings, 1994; Slatkin & Lande, 1976). Conversely, a less optimal environment reflects an environment where phenotypes are far from the phenotypic optimum. Since selection typically does not take place in such environments, relatively higher expression of V_e_ might then be expected.

We also consider each trait or trait group as independent, an assumption that is likely to be violated. Genetic correlations between the means of different traits that influence evolution of trait means can vary across environments (Rauw et al., 1998; Sgrò & Hoffmann, 2004), and we know very little of the potential for genetic correlations between micro‐environmental variances across traits, or even between micro‐environmental variance of one trait and mean of another trait (Mulder, Hill, & Knol, 2015).

Another assumption in our interpretation is that similar modes of selection (breeding goals) occur across countries and in different cattle breeds. Beef and dairy cattle are obviously selected for different traits, and within breeds, the emphasis of breeding programs can differ, such as for increased milk yield in one instance and increased milk fat percentage in another instance, leading to country‐specific breeding goals and production levels (http://www.interbull.org/ib/geforms). However, while the weight given to different production traits in a merit index will differ strongly between dairy and beef cattle, the general breeding goals are in the same direction in both groups (Cunningham, 1983; Miglior et al., 2017), and across breeds and countries, there is an overall focus on mild‐mannered animals adapted to agricultural environments. For breeds exposed to a minor increase in the strength of selection for a given trait, phenotypes far from the optimum will be more disadvantageous, and a steeper decrease in V_e_ is therefore expected as the trait value approaches the optimum. With different strengths of selection acting on traits across breeds and countries, this may introduce some variance into the dataset, but the expectation of a reduction in V_e_ near the optimum still holds and should not affect the patterns detected. We suspect that livestock data constitute an important source of information, which complements theoretical and experimental approaches for answering key evolutionary questions.

## 5 CONCLUSIONS

We quantified the environmental dependence of micro‐environmental variance, with the aim of relating these patterns to past selection on trait means in optimal environments. Using livestock data, we found patterns of a decrease in CV_R_ in the environments where we expected strongest historical selection, a pattern that may reflect selection. Our findings send a clear signal to animal breeders and evolutionary biologists that micro‐environmental variance likely evolves in response to selection on a trait's mean. Evidence for genetic variation underlying micro‐environmental variance has been reported from several studies (Hill & Mulder, 2010; Ibáñez‐Escriche, Sorensen, Waagepetersen, & Blasco, 2008). This suggests that future selection targeting micro‐environmental variance could be successful in increasing animal robustness and welfare, and lead to more uniform production within environments (see also Blasco et al., 2017; Mulder et al., 2007; Mulder et al., 2008). Similar recommendations have recently been put forward for macro‐environmental variance (Mulder, 2016). Thus, our approach and findings constitute examples of how evolutionary theory can guide applied sciences such as animal breeding while producing important evolutionary insights.

## CONFLICT OF INTEREST

None declared.

## Data Availability

Data for this study are available in the supplementary information.
