# Supplementary material for: Patterns of environmental variance across environments and traits in domestic cattle
Source: Evol Appl. 2020 Mar 6;13(5):1090–102. doi: 10.1111/eva.12924 (PMC7232762; doi:10.1111/eva.12924)

Supplementary material for

Patterns of environmental variance across environments and traits in domestic cattle

Excluded data

V_P estimates of age at first calving (days) from Muasya et al. (2014) were 100 times smaller than the two other studies providing data on this trait. We excluded the values from this reference because it suggests that measures were made on different scales or for different traits with the same name.

Tables

Table S1. Classification of countries into three levels of environmental quality. Excel file.

Table S2. Trait definitions and trait grouping into evolutionary categories. Excel file.

Table S3. Testing the effect of environmental quality on lnCVR in each trait category using the R-package 'MCMCglmm'. Excel file.

Table S4. Estimates of CV_R and CV_{WI} obtained from the literature survey. Excel file.

Figures

Figure S1. Comparison of the two measures of favourability (trait mean and environmental quality) in studies where traits of the same breeds were measured at several quality levels.

Figure S2. Distribution of estimates from different trait groups across cattle breeds.

Figure S3. Association between the standard deviation (the square-root of V_e) and mean trait value.

Figure S4. Comparison of CV_R between free-ranging and confined environments.

Figure S5. CV_R across traits according to the quality of the environment.

Figure S6. Summary of CV_R estimates across traits within each of five trait categories.

Figure S1. Comparison of the two measures of favourability (trait mean and environmental quality) in studies where traits of the same breeds were measured at several quality levels. For each study the trait mean was averaged across estimates, and connected by a dashed line. When applying a Wilcox signed rank test to assess whether the trait mean was higher in high quality environments (low vs intermediate and high vs intermediate), we reversed the values of age at first calving, as a low mean for this trait is considered favourable.

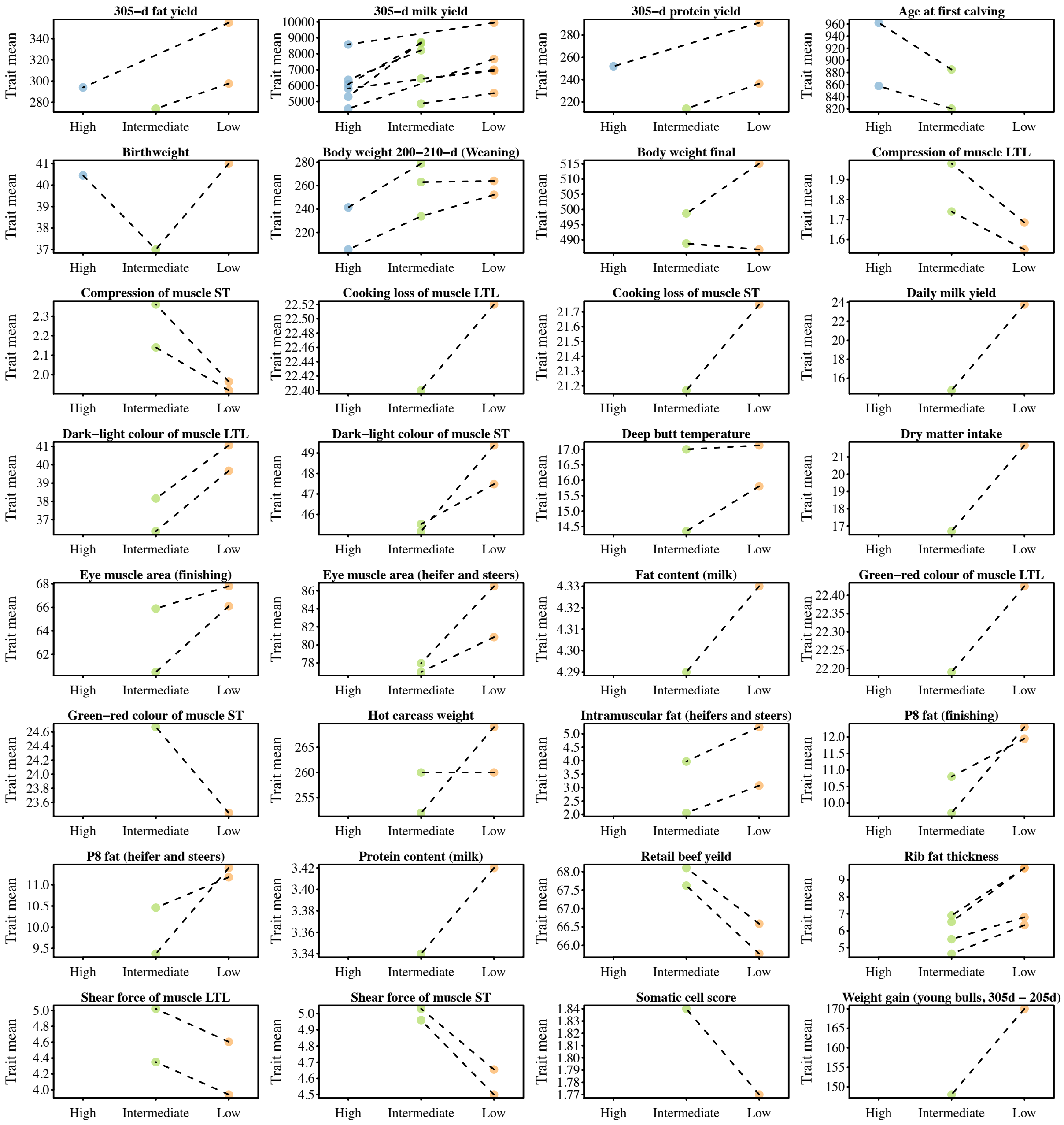

Figure S2. Distribution of estimates from different trait groups across cattle breeds.

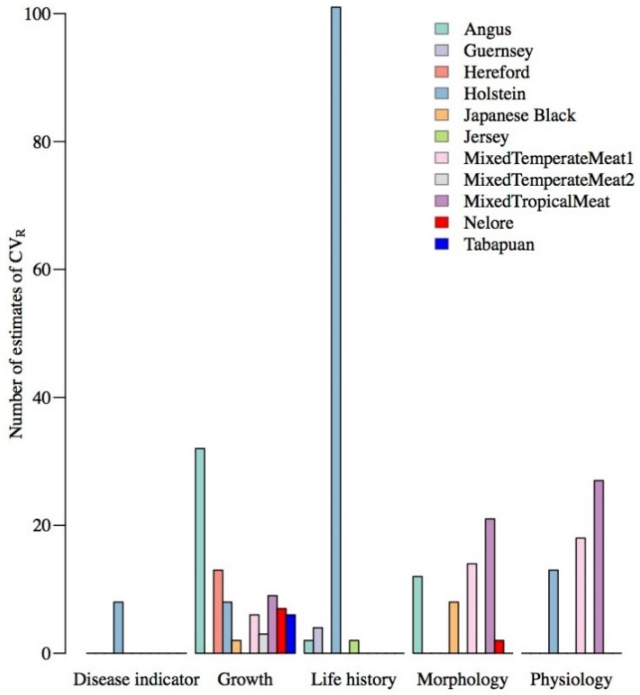

Figure S3. Associations between the standard deviation (the square-root of V_e) and mean trait value. Most traits were represented with only a few estimates making it difficult to infer if there was a correlation and we therefore merged traits that were on the same scale. This was typically traits which represented measures of the same trait but at different ages or different body parts. 223 out of a total of 299 estimates were grouped this way and plotted in this figure. The 95% confidence interval of a linear fit relationship is represented by the shaded area.

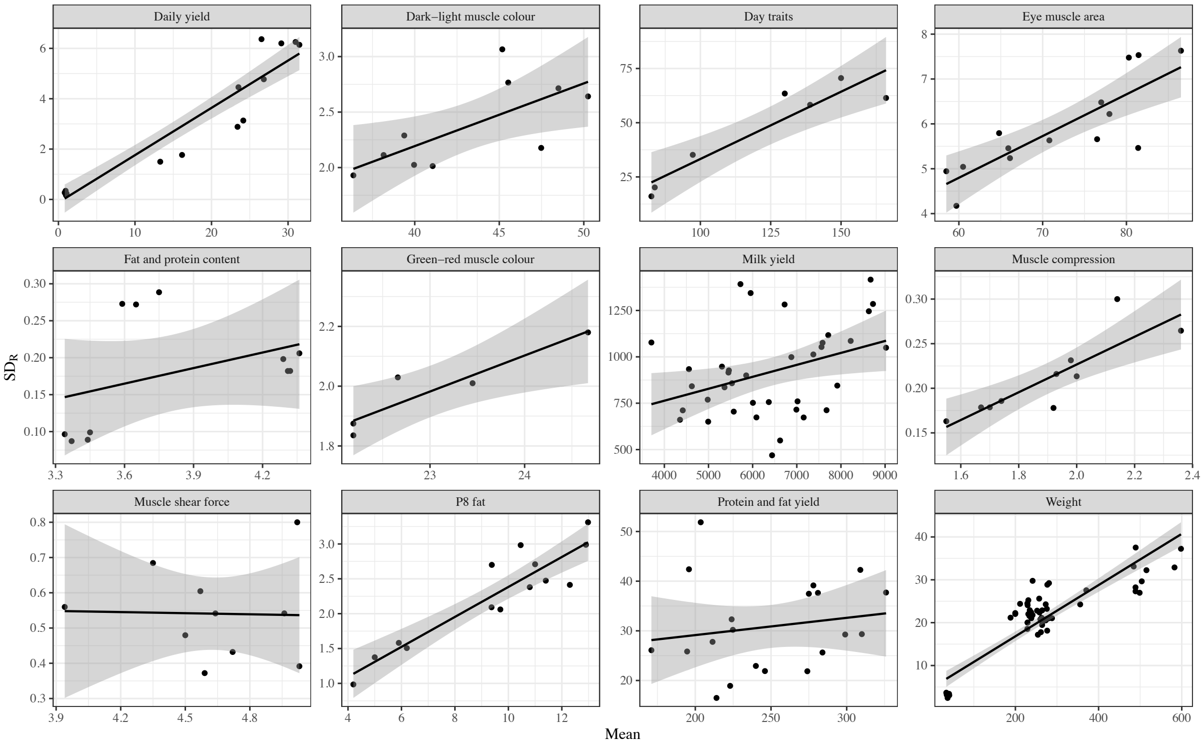

Figure S4. Comparison of CV_R between heterogeneous (free-ranging) and homogenous (confined) environments. Data was obtained from eight studies comparing the performance of cattle in environments contrasted by their difference in rearing. To compare the CV_R between the two environments within a study, we calculated the coefficient of variation ratio ($lnCV_{R\ homogeneity}$), for which negative values indicate lower CV_R in the more homogenous environment *and vice versa*.

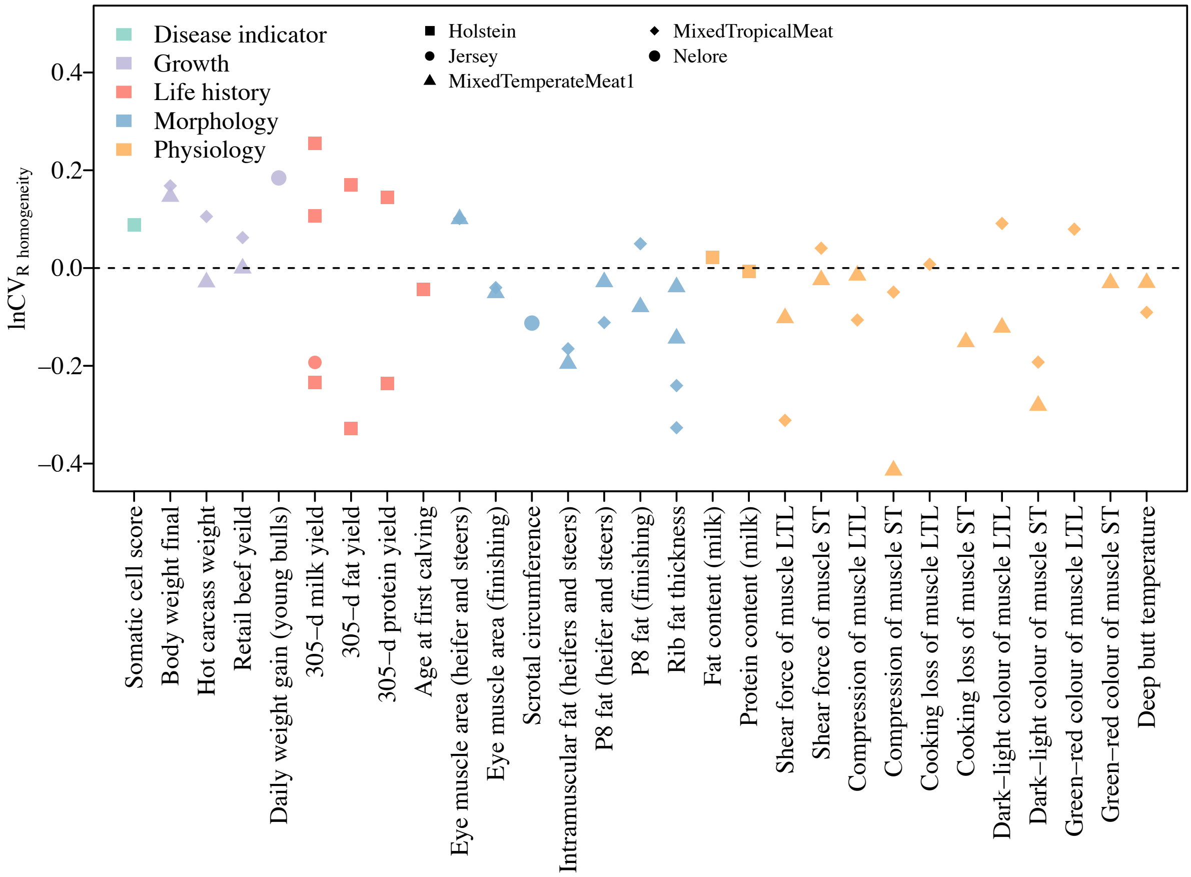

Figure S5. CV_R across traits according to the environmental quality.

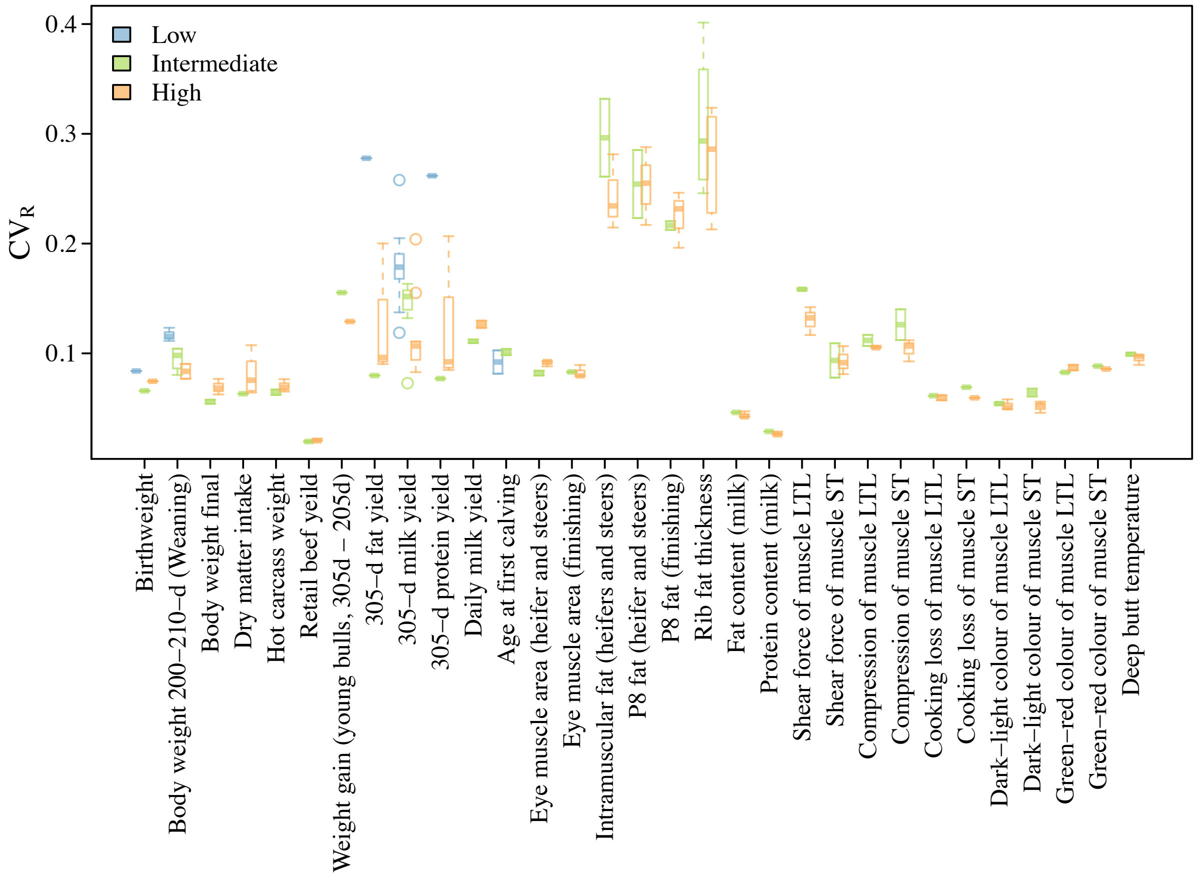

Figure S6. Summary of CV_R estimates across traits within each of five trait categories. CV_R is a proxy of V_e and was estimated as the coefficient of residual variance that includes all terms except V_A. For some traits, estimates of repeatability were available, which allowed us to calculate the within individual environmental variance (CV_{WI}). The colouring represents the five trait categories.

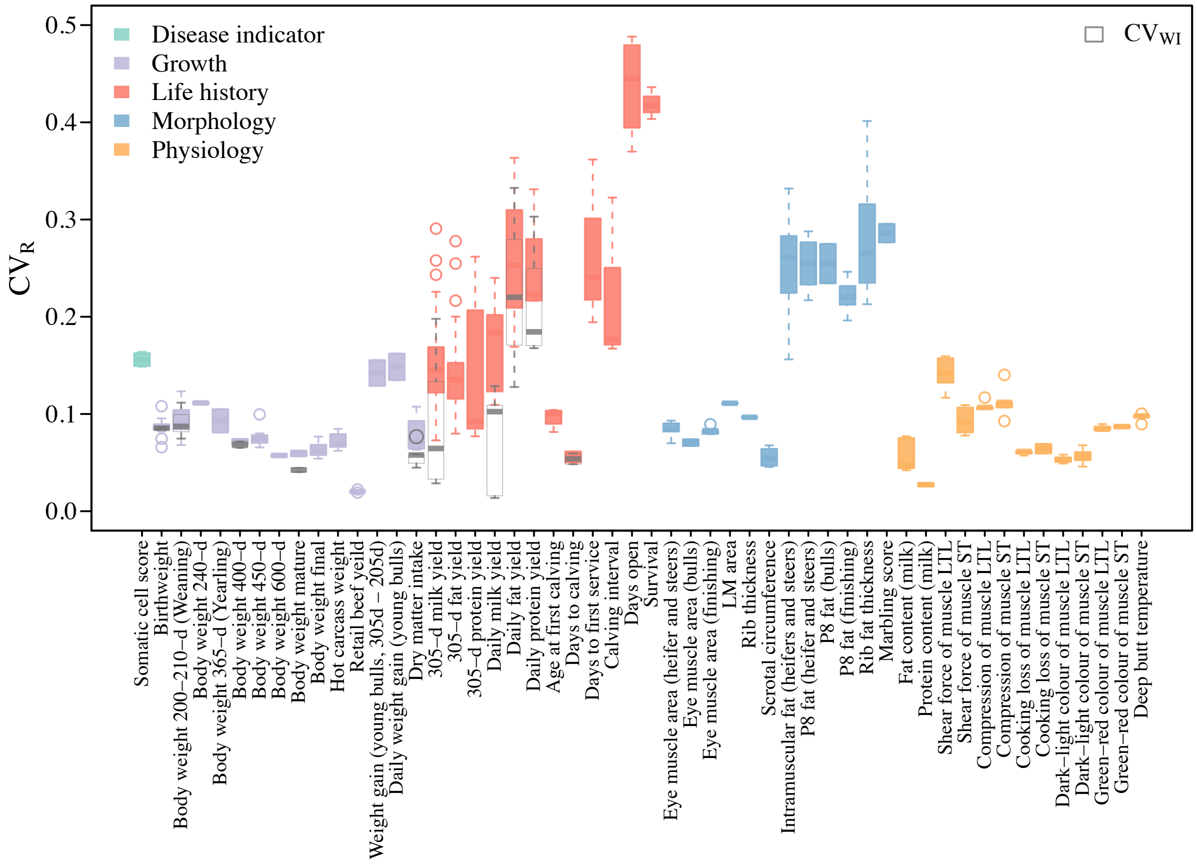

Supplement: Supplementary file 1 [file EVA-13-1090-s001.pdf]
